# 3D evaluation of the morphological and volumetric changes of the tongue and oral cavity before and after orthognathic surgery for mandibular prognathism: a preliminary study

**DOI:** 10.1186/s40510-020-00331-7

**Published:** 2020-08-28

**Authors:** Airy Teramoto, Shoichi Suzuki, Norihisa Higashihori, Naoto Ohbayashi, Toru Kurabayashi, Keiji Moriyama

**Affiliations:** 1 Department of Maxillofacial Orthognathics, Graduate School of Medical and Dental Sciences, Tokyo Medical and Dental University, 1-5-45 Yushima, Bunkyo-ku, Tokyo, 113-8549, Japan; 2 Department of Oral and Maxillofacial Radiology, Graduate School of Medical and Dental Sciences, Tokyo Medical and Dental University, 1-5-45 Yushima, Bunkyo-ku, Tokyo, 113-8549 Japan

**Keywords:** Vinyl polysiloxane, Tongue volume, Oral cavity capacity, 3D images, Orthognathic surgery

## Abstract

**Background:**

The volumetric ratio of the tongue to the oral cavity has been recognized to be one of the important factors for the maintenance of stable occlusion. Oral cavity capacity is changed after orthognathic surgery in patients with mandibular prognathism; however, the volumetric changes of the oral cavity including the tongue before and after surgery have not been analyzed. The purpose of this study was to evaluate the morphological and volumetric changes of the tongue and oral cavity following orthognathic surgery using a newly developed vinyl polysiloxane impression method.

**Materials and methods:**

The study was performed in fifteen subjects who underwent surgical orthognathic treatment. Impressions of the tongue together with the oral cavity were obtained before orthognathic surgery and 1, 3, and 6 months after orthognathic surgery. These impression patterns were scanned using cone-beam computed tomography (CT), and three-dimensional (3D) images of the oral cavity including the tongue, and the upper and lower dental arches were reconstructed. The morphological and volumetric changes in the oral cavity capacity and the tongue volume were examined.

**Results:**

The volume of the tongue with the volume of the oral cavity decreased after orthognathic surgery. There was a correlation between the decrease in the oral cavity capacity and tongue volume. The volumetric ratio of the tongue to the oral cavity seems to be maintained before and after orthognathic surgery.

**Conclusion:**

VPS method, free from radiation exposure, may be useful for investigating the morphological and volumetric changes of the tongue and oral cavity, which may possibly influence the stability of the dental arch and occlusion during surgical orthodontic treatment.

## Background

According to the equilibrium theory, the ratio of the tongue volume (TV) to the oral cavity capacity (OCC) might be an important factor for the maintenance of a stable dental arch shape and proper occlusion [1]. In orthodontic treatment, procedures such as retraction of the anterior teeth or expansion of the dental arches may result in volumetric changes in the oral cavity [2]. In patients with facial deformities, jawbone displacement changes the morphology of the oral cavity, including constriction of the dental arch forms [3]. Wickwire et al. speculated that the tongue position or function, or both, compensate for changes in the oral environment, and that the tongue will adapt to the size of the mandibular arch containing it [4]. This possibly explains why significant volumetric changes in surgical orthodontic treatment cases show a stable occlusion after orthognathic surgery [5, 6]. 

Measurements of changes in the tongue area after orthognathic surgery have been reported in previous papers. Tseng et al. investigated the correlation between postoperative stability and changes in the tongue area after the treatment of mandibular prognathism by using lateral cephalograms. The measured tongue area on the lateral cephalogram corresponded to the area upper to the most superior and anterior points of the hyoid bone, and the most prominent point of the mandibular symphysis posterior border. There was no significant correlation between postoperative skeletal relapse and a change in tongue area. Based on their findings, Tseng et al. suggested that the tongue and hyoid bone changes its position and patterns, together with the displacement of the mandibular position by orthognathic surgery [7]. Jakobsone et al. evaluated the area and volumetric changes in the upper airway and tongue volume after bimaxillary correction of Class III malocclusion by computed tomography (CT). The tongue was measured based on the anatomical criteria; the muscles forming the floor of the mouth were excluded from the calculation. The postoperative tongue volume was not significantly different [8]. To better understand the influence of the tongue on the stability of the occlusion after orthognathic surgery, it is important to measure the volume of the oral cavity and tongue to determine the volumetric balance between OCC and TV. The OCC and TV have been measured accurately by cone-beam computed tomography (CBCT) examinations [9–11]. However, CBCT examinations involve exposure to radiation. Moreover, assessments of the volumetric changes of OCC and TV during orthognathic treatment involve repeated examinations, and the use of multiple CBCT examinations for this purpose would not be ethical due to the probable negative health effects.

Takada, Tamari, and Oliver used an alginate impression method to measure tongue volume [12–14]. In this method, when the tongue impression was taken, the patients were asked to protrude the tongue from the mouth [14]. The impression of the tongue was obtained by taking an impression pattern, and the volume was measured by cast models using the Archimedes principle [12]. This method is simple to perform and is free from radiation exposure. However, the degree of protrusion of the tongue varies among individuals depending on the patient’s skill levels, and the relationship between tongue volume and the oral cavity was not measured.

Vinyl polysiloxane paste produces highly accurate impressions because it reproduces fine surface details and shows elastic recovery, adequate tear strengths, and exceptional dimensional stability [15]. The viscosity of VPS allows it to flow into narrow spaces, such as the space between the tongue and the bottom of the oral cavity. The VPS impression pattern can be scanned and analyzed three-dimensionally (3D) accurately with a digital program [16] to determine OCC and TV.

In this study, we developed an impression method using vinyl polysiloxane paste with a customized tray to obtain OCC, TV, and dental arches in occlusion. By using this VPS impression method (VPS method), examinations of the morphological and volumetric analysis of the tongue and oral cavity were performed at any time during surgical-orthodontic treatment without radiation exposure. The null hypothesis assumed that the volumetric ratio of the tongue to the oral cavity will change before and after orthognathic surgery. The purpose of this study was to evaluate the morphological and volumetric changes of the oral cavity and tongue before and after surgery using the VPS method.

## Materials and methods

### Subjects

Fifteen Japanese subjects (4 males, 11 females) with a mean age of 26.11 ± 6.32 years ranging between the ages of 19.74 and 32.65 years. All subjects met the following criteria: these patients attended the orthodontic clinic of the Tokyo Medical and Dental University and were scheduled to undergo orthognathic surgery for the correction of skeletal Class III malocclusion, had no history of trauma or other congenital craniofacial abnormalities, no congenital missing teeth, no abnormal oral function, no anterior or posterior open bite, no history of maxillofacial surgery, and a mandibular shift less than 3.0 mm. The study was approved by the Ethical Committee of the Tokyo Medical and Dental University (No. D2017-009). Informed consent was obtained from all subjects.

According to the lateral cephalometric measurements obtained before orthognathic surgery, the mean overjet was − 5.63 ± 3.49 mm, mean overbite was 1.43 ± 1.22 mm, and mean ANB angle was − 3.92 ± 2.02°. The surgical procedures in all cases consisted of bilateral sagittal split osteotomy and Le Fort 1 osteotomy. The mean displacement in the mandibular setback at the point pogonion was 7.61 ± 2.33 mm; for the maxilla, ANS anterior displacement was 1.76 ± 1.98 mm, and PNS impaction was 2.06 ± 1.90 mm.

### VPS method

**Fig. 1:**
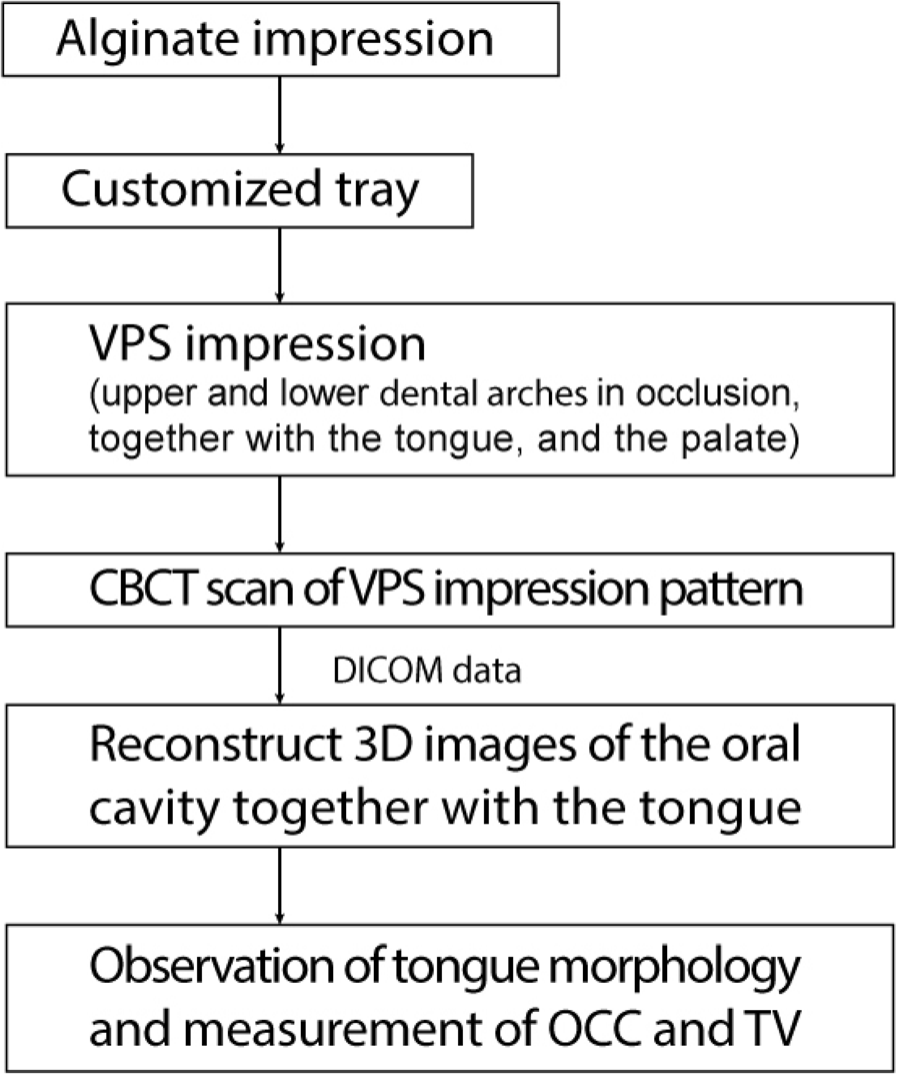
Methodology flow chart. An alginate impression was taken to make a customized impression tray. The impression of the upper and lower dental arches in occlusion, together with the tongue, and the palate were taken using VPS impression paste. Then, VPS impression pattern was scanned by cone-beam CT, and the DICOM data was obtained. The 3D images of the oral cavity including the tongue were reconstructed from the DICOM data. Observation of tongue morphology and measurements of OCC and TV were performed

Figure 1 shows the flowchart of the method adopted in this experiment. VPS method was used to obtain the impression of the tongue together with the oral cavity, which included the upper and lower dental arches in occlusion, tongue, and palate. The procedure for VPS method was as follows.

**Fig. 2:**
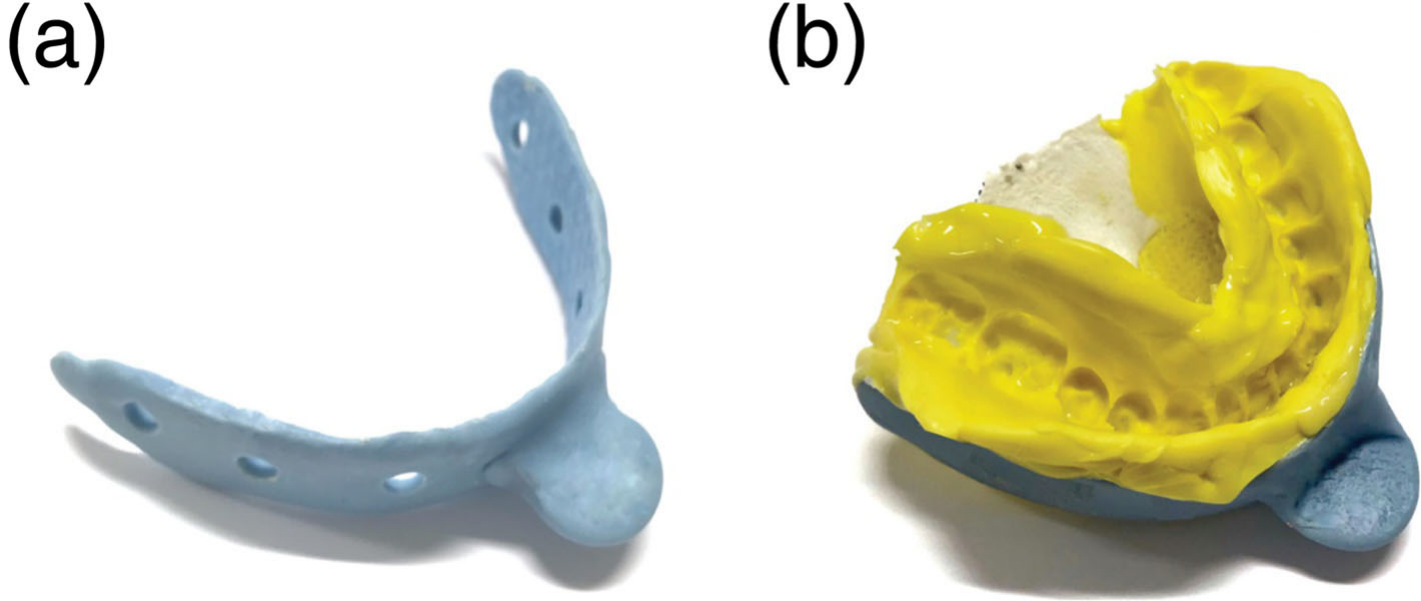
VPS impression pattern. **a** The customized tray used to take VPS impression pattern passing along the buccal side of the upper and lower arches in a “U” form to provide support to the impression. **b** The lower view of VPS impression pattern together with the customized tray and the lower dental arch together with the tongue shape can be observed

First, an alginate impression of the upper and lower dental arches was taken for each subject, and a dental cast was obtained. Using the dental cast in occlusion, a customized tray was made with a thin layer of resin (Ostron II; GC Corporation, Tokyo, Japan) (Fig. 2a). The same examiner made all the impressions. Two types of VPS materials with different viscosities were used to obtain a precise impression pattern with low potential for tearing and distortion by using Correct Plus Thick-n-Thin Light Body (Pentron Clinical, Orange, CA, USA) and Imprint 3 Light Body (3M ESPE, Maplewood, MN, USA). Before the experiment, to familiarize the subject with the impression paste and maintain the tongue position within the impression material, some VPS material was inserted into the oral cavity and the space surrounding the tongue. Then, the subjects were instructed to practice putting the tongue at rest position with some impression paste inside the mouth, with teeth in occlusion. This position was rehearsed several times before making the impression pattern.

To obtain VPS impression pattern, Imprint material was first inserted on the floor of the mouth, after which the Correct Plus material was diffused on the dorsum of the tongue. The imprint material was placed on the occlusal surfaces of the lower teeth and over the entire surface of the tongue body, and the customized tray was then inserted. Each subject practiced biting in the habitual occlusal position, relaxing, setting the tongue in the resting position and putting the tip of the tongue on the lingual surface of the lower incisors. After VPS was catalyzed, the impression pattern was removed from the subject’s mouth (Fig. 2b).

We repeated this VPS method for all subjects before orthognathic surgery (T0), 1 month after orthognathic surgery (T1), 3 months after orthognathic surgery (T2), and 6 months after orthognathic surgery (T3). At each time point, VPS method was taken in duplicate for the same subject on the same day, and the volumetric average was obtained.

### Segmentation and measurement

**Fig. 3:**
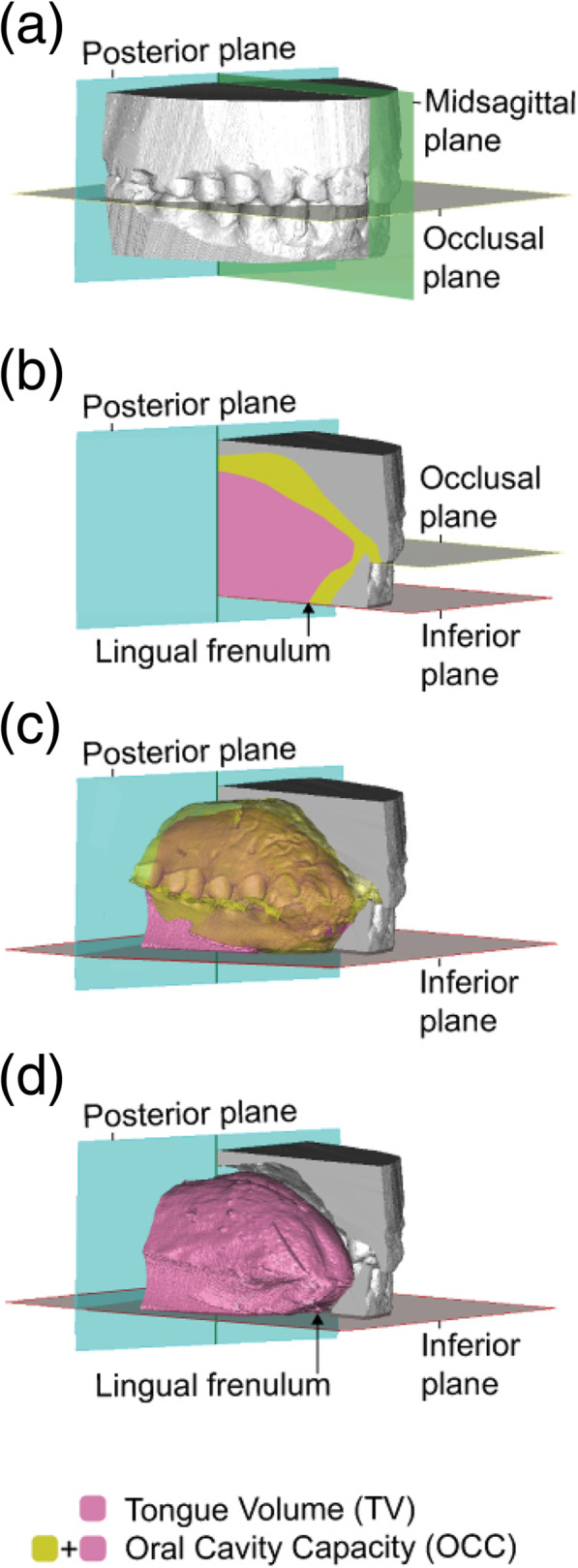
Definition of the measurement area of OCC and TV. **a** The occlusal plane (gray) was defined as the plane passing through the central cusp of the lower first molars and the incisal edge of the right central incisor. The posterior plane (blue) was defined as the plane passing through the distal cusp of the upper second molar perpendicular to the occlusal plane. **b** The inferior plane (red) was defined as the plane passing through the lingual frenulum parallel to the occlusal plane. **c** The oral cavity capacity (OCC) was defined as the space surrounded by the upper and lower dental arches, palate, posterior plane, and inferior plane (yellow). **d** Tongue volume (TV) was defined as the area of the tongue within OCC (pink plus yellow)

The impression pattern was scanned using cone-beam CT (CBCT) (FineCube XP62 system; Yoshida Dental Manufacturing, Tokyo, Japan) with the following parameters: voltage—90 kV, current—4 mA, scan time—8.6 s, field of view—81 × 74 mm, and isotropic resolution—0.2 mm. Digital Imaging and Communications in Medicine (DICOM) data was imported into the SimPlant Crystal software (Materialise Dental, Leuven, Belgium), providing 512 image slices of 0.147 mm thickness. To create 3D objects, we first transformed the impression pattern into a 3D object and then subtracted the tongue space from the 3D impression pattern and reconstructed the tongue, palate, and upper and lower dental arches. For volume analysis, the occlusal plane was defined as the plane passing through the central cusp of the lower first molars and the incisal edge of the right central incisor. The posterior plane was defined as the plane passing through the distal cusp of the upper second molar perpendicular to the occlusal plane (Fig. 3a). The inferior plane was defined as the plane passing through the lingual frenulum parallel to the occlusal plane (Fig. 3b). The OCC was defined as the space surrounded by the upper and lower dental arches, palate, inferior plane, and posterior plane (Fig. 3b, c). Tongue volume (TV) was defined as the tongue region within OCC (Fig. 3b, d). The outlines of the tongue and oral cavity were traced by the program and checked by the same examiner. After reconstructing 3D images, OCC and TV were measured using the 3D image analysis software SimPlant. The TV/OCC ratio at each time point was calculated from the values of OCC and TV obtained from each subject. The changes in TV/OCC ratio during the experimental period were evaluated. The same examiner performed all the VPS methods, the segmentation, and the measurement of the data.

### Method error and intra-subject error

In order to evaluate the reliability of the method, VPS method was applied to an acrylic cuboid cube. The impression of the acrylic cuboid cube (30 mm × 30 mm × 61 mm) was taken with the VPS method seven times by the same examiner. Each impression pattern was scanned by CBCT. Then, a 3D image of the acrylic cuboid cube was reconstructed, and the volume of the 3D image of the acrylic cuboid cube was calculated. The volume of the 3D images of the acrylic cuboid cube was compared with the volume of the real acrylic cuboid cube, and the value of the difference and the percent difference was calculated.

To assess the intra-subject error, VPS method was repeated ten times over 14 days to the same subject by the same examiner. The coefficient of variation was used to estimate the intra-subject error.

### Statistical analysis

The statistical analysis was performed using GraphPad Prism for Windows, version 6.0 (GraphPad Software, Inc., La Jolla, CA, USA). Holm-Sidak’s multiple comparisons test and two-way ANOVA test with a post hoc Sidak correction method were performed to determine significant differences in OCC and TV before and after orthognathic surgery. The OCC and TV reductions after orthognathic surgery were measured using linear regression analysis. The TV/OCC ratios from T0 to T3 were analyzed using one-way ANOVA and Geisser-Greenhouse correction. The results were considered significant at *P* < 0.001.

## Results

### Morphological observations in 3D images of the oral cavity and tongue

**Fig. 4:**
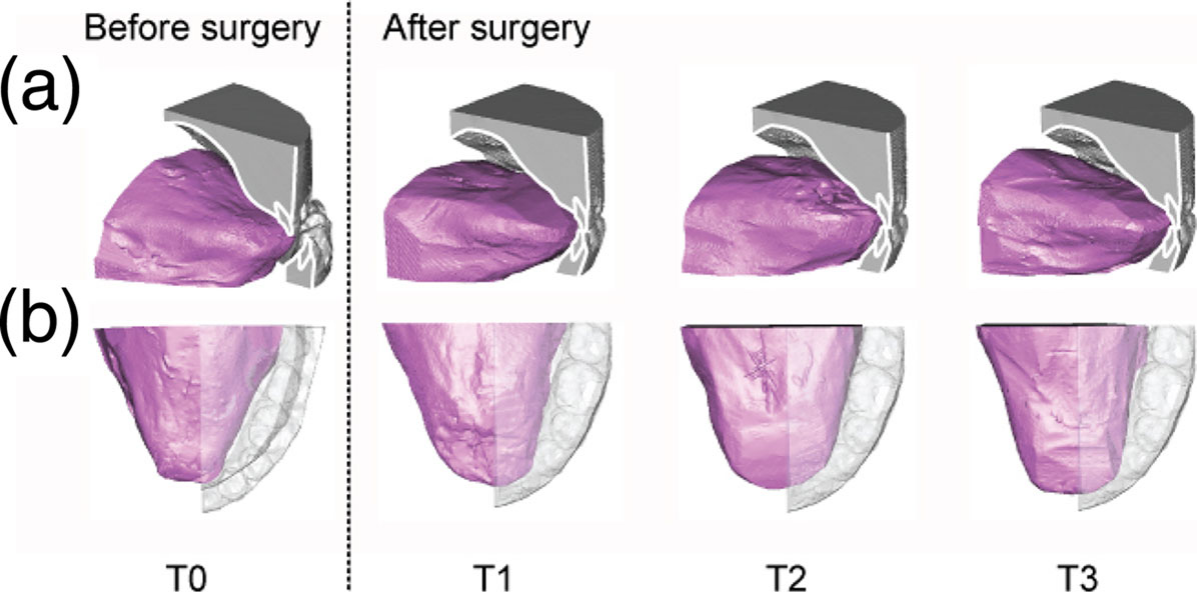
3D images of the half model reconstructed from the DICOM data of VPS impression pattern. The changes in the overjet value and the morphological and positional changes of the tongue inside the oral cavity at the time points T0, T1, T2, and T3 from two different 3D images views. **a** Oblique lateral view. **b** Upper occlusal view

In this study, VPS method was used to analyze the oral cavity and tongue in fifteen subjects with mandibular prognathism before and after orthognathic surgery. VPS impression patterns of the oral cavity were scanned by CBCT and transformed into digital data. From the CBCT data, 3D images of the tongue and oral cavity were reconstructed. 3D images at T0, T1, T2, and T3 from two different views for a subject can be seen in Fig. 4. The oblique lateral views with the half model (Fig. 4a) and the upper-occlusal views with a translucent half model (Fig. 4b) were also obtained.

As shown in Fig. 4a, before orthognathic surgery (T0), the overjet had a negative value, and after orthognathic surgery (T1, T2, and T3), the overjet changed to a positive value. The changes in the overjet value revealed displacement of the jawbone after orthognathic surgery. In the oblique lateral view at T0, the anterior part of the tongue dorsum appeared to be in a slightly lower position, although the posterior part of the tongue dorsum stayed in a higher position. On the other hand, at T1, T2, and T3, the body of the tongue, including the anterior part of the tongue, seems slightly swollen and lifted. Particularly at T2 and T3, the anterior part of the tongue showed upper positioning, and the antero-posterior tongue dorsum line appeared to be in a horizontal position (Fig. 4a). From the upper occlusal view, the posterior part of the tongue in T0 appeared to spread laterally, whereas the anterior part of the tongue appeared narrow, and the outline of the tongue appeared to be triangular. At T1, T2, and T3, the width of the anterior part of the tongue seemed to increase, and the outline of the tongue appeared to show a parabolic shape (Fig. 4b).

### Volumetric changes in OCC and TV

**Fig. 5:**
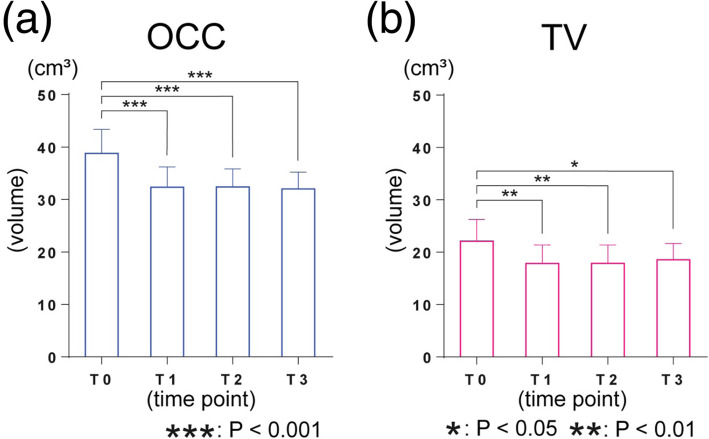
Value of OCC and TV at each time point. **a** The OCC values at T1, T2, and T3 were significantly smaller than T0 (T1, T2, T3: *P* < 0.001). **b** The TV values at T1, T2, and T3 were significantly smaller than T0 (T1, T2: *P* < 0.01, T3: *P* < 0.05)

**Fig. 6:**
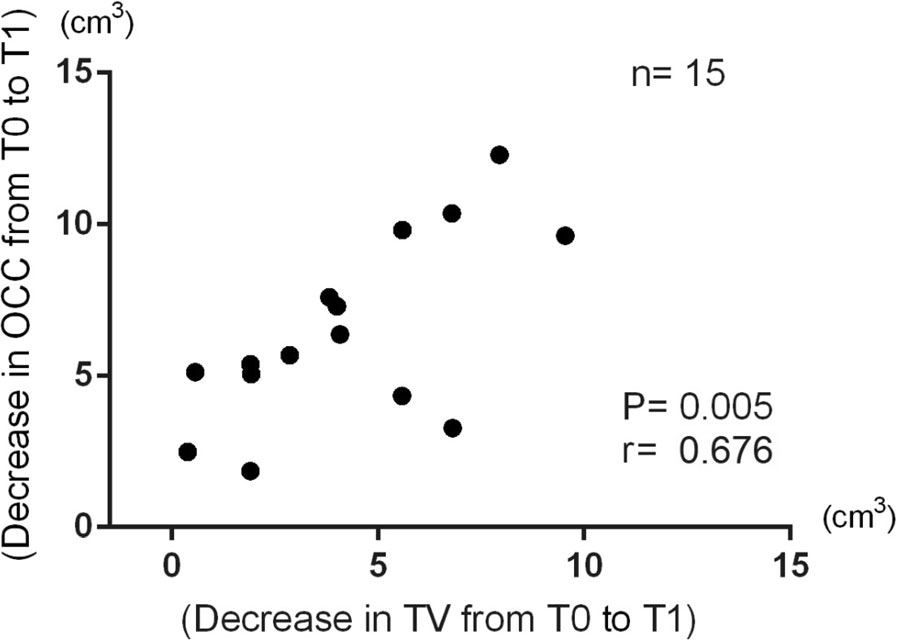
The relation between the decrease in OCC and TV. The decrease in OCC showed a positive correlation with the decrease in TV from T0 to T1 after using the linear regression analysis (*P* = 0.005, *R* = 0.676)

The mean of OCC was 38.79 ± 4.55 cm^3^ at T0, 32.35 ± 3.84 cm^3^ at T1, 32.37 ± 3.48 cm^3^ at T2, and 32.01 ± 3.20 cm^3^ at T3, and the mean of TV was 22.09 ± 4.18 cm^3^ at T0, 17.85 ± 3.54 cm^3^ at T1, 17.92 ± 3.34 cm^3^ at T2, and 18.57 ± 3.31 cm^3^ at T3. Holm-Sidak’s multiple comparison showed that OCC decreased significantly from T0 to T1, from T0 to T2, and from T0 to T3. However, the volumetric difference of OCC from T1 to T2 and from T2 to T3 was not significant. The average decrease in OCC following orthognathic surgery was 16.35%. In addition, TV decreased significantly from T0 to T1, T0 to T2, and T0 to T3. However, the volumetric difference of TV from T1 to T2 and from T2 to T3 was not significant (Fig. 5). The volumetric changes in OCC and TV in 15 subjects during the experimental period were assessed using a two-way ANOVA test with a post hoc Sidak correction. The interaction between OCC and TV was not significantly different from T0 to T3. For the relationship between the decreased value of OCC and TV from T0 to T1, the results revealed a correlation between the variables (*P* = 0.005, *R* = 0.676). A well-balanced decrease in sync with OCC of TV was observed (Fig. 6).

### Changes in TV/OCC ratio

**Fig. 7:**
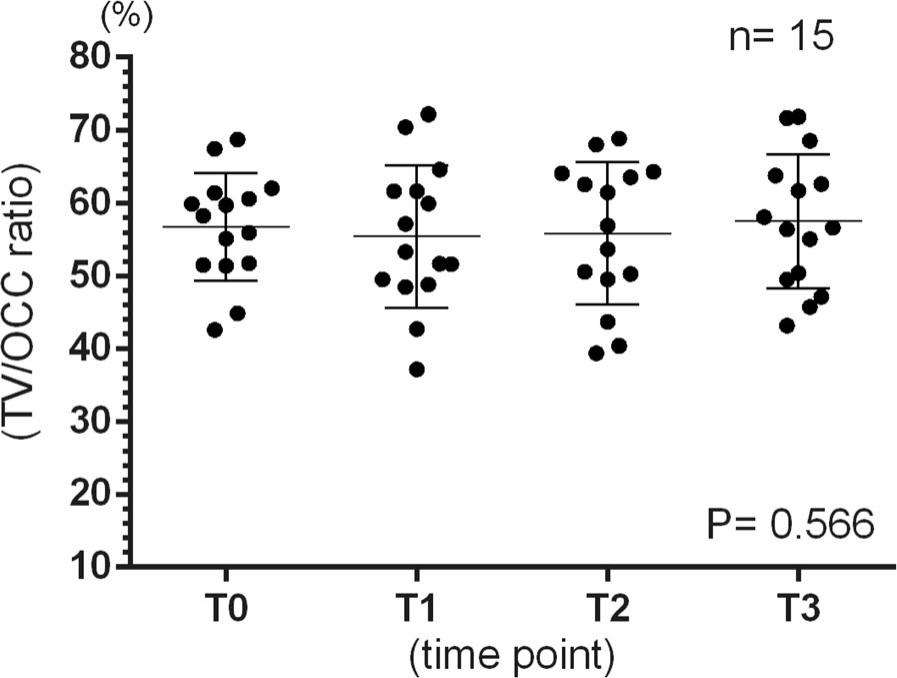
The TV/OCC ratio of all subjects. The mean ± S.D. of TV/OCC ratio was 56.75 ± 7.43% at T0, 55.41 ± 9.80% at T1, 55.84 ± 9.83% at T2, and 57.50 ± 9.21% at T3. Repeated one-way ANOVA and Geisser-Greenhouse correction were assessed, and no significant difference was observed among the four time points (*P* = 0.566)

TV/OCC ratio was measured, and the mean TV/OCC ratio was 56.75 ± 7.43% at T0, 55.41 ± 9.80% at T1, 55.84 ± 9.83% at T2, and 57.50 ± 9.21% at T3. Repeated one-way ANOVA test with Geisser-Greenhouse correction was performed to assess these findings. No significant difference was found among the four time periods (*P* = 0.556; Fig. 7).

**Fig. 8:**
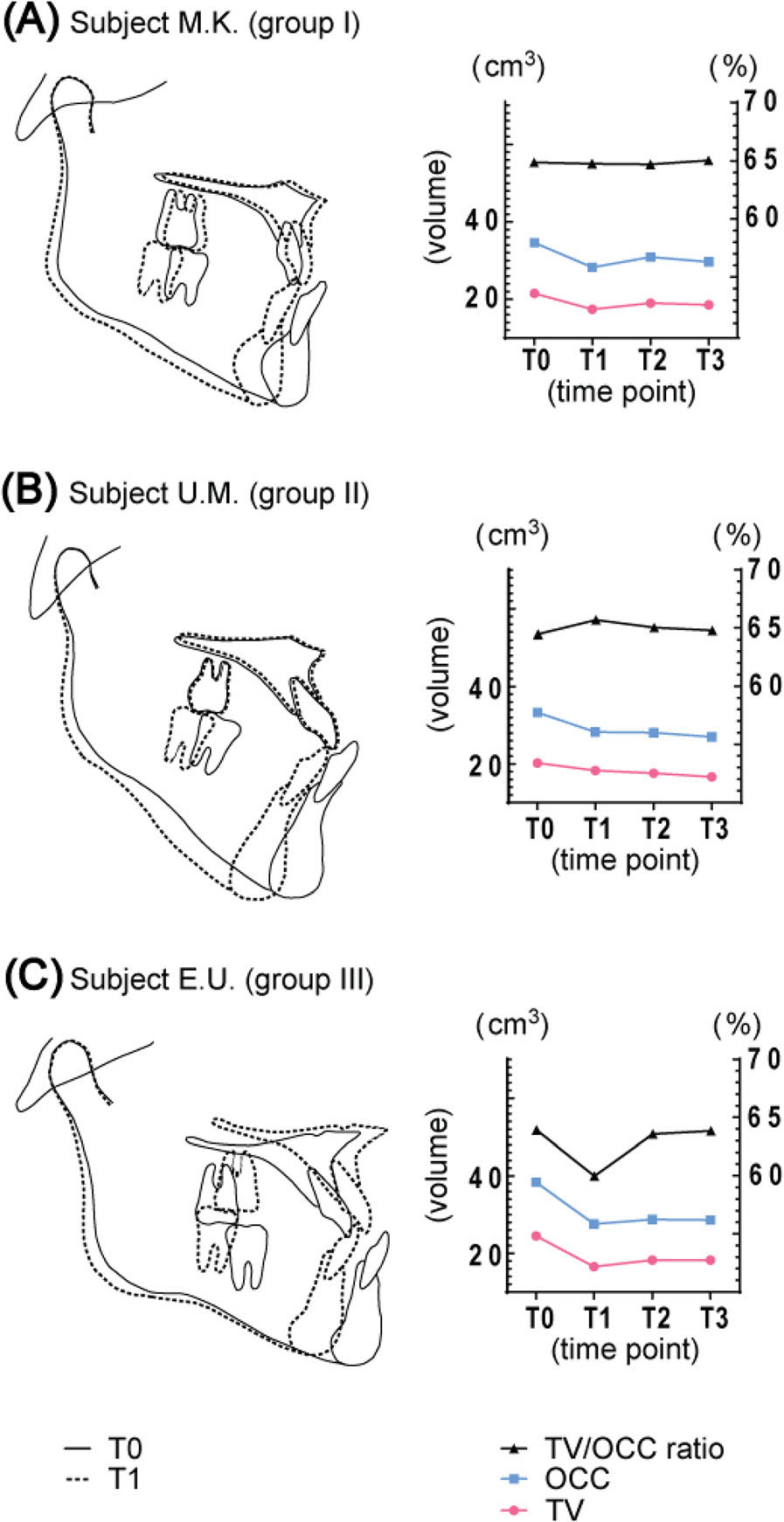
Superimposition of the cephalogram tracing and transition of TV, OCC, and TV/OCC ratio. **a** Group I representative—Subject M.K. (female; 35 years, 2 months). Superimposition of cephalogram tracing between T0 (solid line) and T1 (dotted line). The value of the change of TV/OCC ratio from T0 to T1 and from T1 to T3 was small, and the transition of TV/OCC ratio was stable during all the experiment period. **b** Group II representative—U.M. (female; 25 years, 6 months). Superimposition of cephalogram tracing between T0 (solid line) and T1 (dotted line). Although the value of the change of TV/OCC ratio from T0 to T1 increased, the value from T1 to T3 got closer to TV/OCC ratio value at T0. **c** Group III representative—subject E.U. (male; 26 years, 8 months). Superimposition of cephalogram tracing between T0 (solid line) and T1 (dotted line). Although the value of the change of TV/OCC ratio from T0 to T1 decreased, the value of TV/OCC ratio form T1 to T3 got closer to TV/OCC ratio value at T0

The fifteen subjects were classified into three groups based on the value of the changes in TV/OCC ratio from T0 to T1. The subjects in which the change in TV/OCC ratio value was small (within − 2.0 to + 2.0%) were classified as group I. Subjects in which the change in TV/OCC ratio value increased by more than 2.0% were classified as group II. Subjects in which the change in TV/OCC ratio value decreased by less than − 2.0% were classified as group III. From the total fifteen subjects, five subjects belonged to group I, four belonged to group II, and six belonged to group III. Figure 8 shows the superimposition of the cephalometric tracing of the representative subjects belonging to groups I, II, and III before orthognathic surgery and 1 month after surgery, and the transition values of OCC, TV, and TV/OCC ratio during the experimental period. In the subject representing group I, the change in the value of TV/OCC ratio was small from T0 to T1 as well as from T1 to T3, and the transition of TV/OCC ratio was stable during the experimental period (Fig. 8a). In the subject representing group II, although the value of TV/OCC ratio increased greater than 2.0% from T0 to T1, TV/OCC ratio decreased from T1 to T3, and the value of T3 was closer to TV/OCC ratio value at T0 (Fig. 8b). In the subject representing group III, although the value of TV/OCC ratio decreased by less than − 2.0% from T0 to T1, TV/OCC ratio increased from T1 to T3, and the value of T3 was closer to TV/OCC ratio value at T0 (Fig. 8c).

### Method error and intra-subject error

The VPS method error was assessed by comparing the volume of the acrylic cuboid cube and the volume of its 3D image by repeating VPS method seven times. The mean of the difference and the percent difference was 0.11 ± 0.08 cm^3^ and 0.99 ± 0.14%, respectively.

To evaluate the intra-subject error of the method, VPS method was taken for the same subject ten times. The coefficient of variation expressed as a percentage of the mean was 2.10% for OCC and 4.29% for TV. The mean of OCC and TV was 38.36 ± 0.80 cm^3^ (CI, 37.78–38.94 cm^3^) and 23.40 ± 1.00 cm^3^ (CI, 22.68–24.12 cm^3^), respectively (Table 1).

**Table 1:** The mean coefficient of variation from ten times repeated VPS method taken in the same subject

Parameter	Mean ± S.D. (cm^3^)	Coefficient of variation(%)
OCC	38.38 ± 0.80	2.10
TV	23.40 ± 1.00	4.29

## Discussion

### VPS method

To evaluate the reliability of the method, the difference and the percentage difference of the volume of the acrylic cuboid cube and that of its 3D image reconstructed from the digital data of its impression pattern were small (< 1%). This demonstrates the reasonable accuracy of this method for volumetric analysis.

The body of the tongue is composed of voluntary muscles, and its position and morphology continuously change according to its oral function. Its volume may change depending on its morphology and position. Because of tongue mobility, the range of intra-subject error was important to consider when measuring tongue volume. The repeatability of our measurements was assessed by the one-way ANOVA test, and the results showed no significant difference between the measurements. For the intra-subject error, the coefficient of variation for OCC was 2.10% and that of TV was 4.29%. In a previous study involving measurements of the volume of various parts of the human body, Kovacs et al. used an impression method to measure the volume of the breast. Ten measurements were taken for each of the six subjects, and the coefficient of variation of the measured volume was 7.97%. In their thermoplastic cast method, the examiner took the impression of the breast by applying manual pressure. The authors speculated that variations in the pressure applied by the examiner might be a factor that increases the coefficient of variation [17].

### The tongue position

The habitual position of the tip of the tongue has been reported to be at the lingual surface of the lower incisor teeth [18]. In this experiment, the value of TV was determined in this habitual position of the tongue inside the oral cavity. Before making VPS impressions, the subjects were instructed to bite in the habitual occlusal position, relax, set the tongue in the resting position, place the tip of the tongue on the lingual surface of the lower incisors, and remember this position. This tongue position was quite reproducible at each time point in each subject by practicing several times with some amount of the impression paste inside the oral cavity. To minimize intra-subject biases, the positional relationship between the tongue tip and the lingual surface of the lower incisor was carefully confirmed by observing the impression pattern each time. In addition, impressions were taken in duplicate for the same subject on the same day by the same examiner, and their average was used as OCC and TV of the subject.

### Observation of 3D images of the tongue and oral cavity

In this experiment, 3D images of the oral cavity structures were obtained by reconstruction of the images of the impression pattern. These images which included the upper and lower dental arches, the palate, the tongue, and the floor of the mouth are very similar to those obtained by direct CBCT examination [11]. These 3D images were acquired without exposing the patient to radiation. It was very useful because it allowed us to observe the various components of the oral cavity, including the tongue, from various directions. Moreover, we were able to slice the images in any plane. In this experiment, we reconstructed half models to observe the morphological changes in the tongue together with the changes in the occlusion.

The body of the tongue observed from 3D images before orthognathic surgery showed a lower position and laterally spread; after the surgery, the body of the tongue changed to a slightly swollen and lifted position. These observations were consistent with the findings from lateral cephalometric analysis [6, 19]. The changes in the morphology of the body of the tongue were considered to indicate adaptations of the tongue to the changes in OCC because of orthognathic surgery.

### Volumetric analyses of tongue and oral cavity capacity

In previous studies, OCC and TV were measured using various techniques, such as MRI [20], CT [21, 22], CBCT [9–11], and alginate impression material [12–14]^.^ However, the anatomical definitions of the tongue and the measurement areas in these studies were usually inconsistent [20]. We defined TV as the tongue volume within the space surrounding the upper and lower dental arches, palate, and floor of the mouth. This portion of the tongue was measured because this segment may influence the form of the dental arches and occlusion. Although our measurements differ from those in some studies, the areas measured by Uysal et al. and Ding et al. were similar to ours. Ding et al. obtained a tongue value of 47.0 ± 7.08 cm^3^ [11], and Uysal et al. reported values of 31.02 ± 9.75 cm^3^ for males and 28.13 ± 8.54 cm^3^ for females [9]. The values obtained in our study were smaller than those obtained by Uysal et al. and Ding et al. (22.09 ± 4.18 cm^3^). We assumed that several factors might have influenced these differences in volumetric measurements. The landmarks and the measurement areas for TV differed among the studies. In particular, TV measurement area by Ding et al. was larger than our definition. The samples assessed by Ding et al. and Uysal et al. were of subjects with normal occlusion; on the other hand, we measured the tongue volume of subjects with skeletal class III malocclusion. It has been reported that the tongue in angle class III malocclusion is usually found at a lower position [23]. In this study, we may presume that the tongue was displaced slightly lower and backward than in the usual position because of the insertion of the impression paste, which occupies the vacant space inside the oral cavity. The tongue position caused by the method might influence the volumetric differences in tongue volume. To reduce these differences, in the present study, the same impression method was applied by the same examiner for each of the fifteen subjects at four time points; therefore, we considered it acceptable to compare the values of OCC and TV.

### Volumetric changes after surgery

The results showed that the average decrease in OCC was 16.35%. This decrease in OCC was achieved by a 7.61 ± 2.33mm displacement of the mandible setback, 1.76 ± 1.98 mm maxilla advancement, and 2.06 ± 1.90 mm impaction of the maxilla. A correlation was found between the decrease in OCC and TV before and after orthognathic surgery. In this experiment, OCC was defined as the space surrounding the upper and lower dental arches, palate, and the floor of the mouth. Therefore, values indicating a decrease in OCC before and after orthognathic surgery can be considered almost dependent on the displacement of the jawbones. Since the parts of the outer tongue muscle attach directly to the mandible, the tongue mainly displaces its position along with the displacement of the mandible, which could lead to a correlation between the decrease in OCC and TV. Assessment of 3D images revealed that the tongue seemed to move backward and lifted up slightly after orthognathic surgery. These changes in tongue position and morphology might indicate the regulation of TV/OCC ratio maintenance after surgery.

### Changes in TV/OCC ratio

The subjects were classified into three groups based on the values of the changes in TV/OCC ratio from T0 to T1. In the representative cases of groups I, II, and III, we were able to observe the changes in the TV/OCC ratio during the experimental period after the surgery. Although the three groups had different transition patterns of TV/OCC ratio from T0 to T1, their values from T1 to T3 were closer to T0. In addition, we examined the relationship between the length of the maxilla-mandibular displacement and the transition pattern of TV/OCC ratio from T0 to T3, but no relationship could be recognized within the 15 subjects.

Proffit et al. emphasized the importance of neuromuscular adaptation for stability [24], and Nathanson presumed that the tongue would adapt to the size of the mandibular arch containing it [3]. We speculated that the maintenance of TV/OCC ratio after orthognathic surgery might contribute to a stable occlusion after orthognathic surgery, and that the tongue would adapt to the size of the mandibular arch containing it.

### Applications of VPS impression method

VPS impression method was considered to be applicable for investigating the volumetric and 3D morphological changes of the tongue and the oral cavity in the course of the treatment because it is simple and free from radiation exposure. Moreover, this method might provide a way to investigate the dental arch morphology together with the volume of the tongue and oral cavity capacity, although further research is needed in the future. The limitation of this method, however, is that the results may not directly represent the dynamic function of the tongue, but only its static status in the oral cavity. In order to more precisely understand the implication of the tongue in the stability of occlusion, correction and integration of the multi-modality data on tongue morphology and function would be required. In addition, it is necessary to insert impression paste into the oral cavity to make the impression pattern. Therefore, this method may not be used in subjects with a hypersensitive gag reflex and younger subjects, to decrease the burden on the subjects and increase the reliability of the measurements.

In conclusion, the patients with mandibular prognathism showed a decrease in both OCC and TV following orthognathic surgery, and TV/OCC ratio remained unchanged before and after surgery; therefore, the null hypothesis is rejected. VPS impression was considered to be a useful method for evaluating the volumetric and morphological changes of the tongue and the oral cavity which may influence the stability or relapse of tooth alignment and occlusion, without radiation exposure.

## Data Availability

The data supporting the study can be obtained directly from the authors.

## Abbreviations

TV: Tongue volume
OCC: Oral cavity capacity
CT: Computed tomography
CBCT: Cone-beam computed tomography
VPS: Vinyl polysiloxane
VPS method: Vinyl polysiloxane impression method
3D: Three-dimension
T0: Before orthognathic surgery
T1: One month after orthognathic surgery
T2: Three months after orthognathic surgery
T3: Six months after orthognathic surgery
